# Effect of Lactic Acid Bacteria-Derived Postbiotic Supplementation on Tuberculosis in Wild Boar Populations

**DOI:** 10.3390/pathogens13121078

**Published:** 2024-12-08

**Authors:** Maria Bravo, Pilar Gonçalves, Waldo García-Jiménez, María José Montero, Rosario Cerrato, Pedro Fernández-Llario, David Risco

**Affiliations:** 1 Ingulados S.L., Calle Miguel Servet 13, 10003 Cáceres, Spain; maria@ingulados.com (M.B.); pilar@ingulados.com (P.G.); waldo@ingulados.com (W.G.-J.); mjose@ingulados.com (M.J.M.); rosario@ingulados.com (R.C.); pedro@ingulados.com (P.F.-L.); 2 Unidad de Histología y Anatomía Patológica, Departamento de Medicina Animal, Veterinary Faculty, University of Cáceres, Avenida de la Universidad s/n, 10003 Cáceres, Spain

**Keywords:** postbiotic, tuberculosis, wild boar, lactic acid bacteria

## Abstract

The Eurasian wild boar (*Sus scrofa*) is a key wildlife host for tuberculosis (TB) in central and southwestern Spain, posing a challenge to TB eradication in livestock. New strategies, including the use of beneficial microbes, are being explored to mitigate wildlife diseases. This study evaluated the effect of oral supplementation with postbiotic antimycobacterial metabolites produced using Ingulados’ lactic acid bacteria (LAB) collection on TB development in wild boar. A total of 20 game estates in mid-western Spain were divided into two groups: one fed with standard feed containing the postbiotic product and a control group fed without postbiotics. Data were collected from wild boar during hunting events pre- and post-supplementation. The presence of TB-like lesions (TBLLs), lesion severity and seropositivity against *Mycobacterium bovis* were assessed. Postbiotic supplementation led to a 36.87% reduction in TBLLs and a 35.94% decrease in seropositivity. Notably, young wild boar showed a 64.72% reduction in TBLLs and an 81.80% drop in seropositivity, suggesting reduced transmission. These findings support the potential of postbiotics as a safe, feasible and sustainable tool to control TB in wild boar, offering a promising addition to broader TB eradication efforts.

## 1. Introduction

Tuberculosis (TB) is a chronic mycobacterial infection with significant impact on human and animal health and the global economy [1]. Bacteria belonging to the Mycobacterium Tuberculosis Complex (MTC), like *Mycobacterium bovis (M. bovis*) or *M. caprae,* are the causative agents of bovine TB, and their broad host range includes cattle and many other livestock and wild mammals [2,3]. Furthermore, bovine TB is a zoonotic disease, posing a risk to farmers, slaughter operators, or even consumers of products derived from animals infected by the MTC [1,4] Although the disease has been controlled in most developed countries, bovine TB prevalence remains high in areas densely populated with wild reservoirs of *M. bovis* [5,6,7]. Preventing the spread of bovine TB in a multi-host scenario is only attainable if control measures in livestock and wildlife are simultaneously implemented [1,7]; but it raises complex ecological, ethical and economic issues [1,3].

The Eurasian wild boar (*Sus scrofa*) is considered the main wildlife maintenance host in central and southwestern Spain and Portugal, currently impeding decades-long bovine TB eradication programs [3,5,7,8]. Measures focused on controlling TB in wild boar populations are required to eliminate the disease in these regions [1,6]. Some of the most important strategies include preventive actions based on biosecurity measures [3,9], population control through random or selective culling [3,7,9,10] and oral or parenteral vaccination with heat-inactivated *M. bovis* and *M. bovis* Bacillus Calmette–Guérin (BCG) [5,11,12,13]. Similarly, most recent innovative options include vitamin D3 supplementation [14], deworming [15] and vaccination against concomitants [6]. In the majority of cases, besides being technically and economically unfeasible, an intervention in natural ecosystems may be considered controversial [9,10]. Thus, preventive strategies are currently limited, and hence, the need for newer cost-effective and feasible TB control measures is undeniable.

In this sense, several bacterial strains belonging to the microbiome of healthy animals and humans have shown potential activity against *Mycobacterium* spp. [16,17,18]. These beneficial bacteria arise as an environmentally friendly and sustainable alternative in the prevention of disease transmission and dissemination [16,19,20]. Postbiotics are metabolic by-products secreted by beneficial microorganisms and cell wall components released after bacterial lysis which may improve host health due to their anti-inflammatory, immunomodulatory and antimicrobial activity [21,22,23]. Postbiotic metabolites include enzymes, peptides, polysaccharides, short-chain fatty acids and organic acids, among others. In previous in vitro assays, we reported that metabolites from lactic acid bacteria (LAB) antagonize mycobacteria survival by antimicrobial synergistic mechanisms and by influencing phagocytes’ intake [16,20]. Isolated LAB produced two-peptide bacteriocins, a collagen adhesin protein and other cell wall components—like teichoic and lipoteichoic acids—that could act as antimycobacterial agents and innate immunomodulators that delay TB dissemination [16].

The aim of this study was to assess the effect of oral supplementation with postbiotic antimycobacterial metabolites produced by lactic acid bacteria on TB development in wild boar populations to determine if this measure can be a suitable strategy in the fight against TB in this species.

## 2. Materials and Methods

### 2.1. Postbiotic Metabolite Production

The bacterial species chosen for postbiotic production, *Lactiplantibacillus plantarum* CECT9608 and *Lacticaseibacillus paracasei* CECT9610, were isolated from TB-free wild boar populations [16,24]. These strains, part of the Ingulados LAB collection, produce antimicrobial metabolites that inhibit the growth of mycobacteria [16,25]. The postbiotic-derived product is registered (INGUBAL^®^, Ingulados S.L., Cáceres, Spain) as fermented feed containing antimycobacterial metabolites generated in a plant-based matrix during microbial fermentation, under previously described conditions [16,24].

The product is considered safe for use as feed supplement provided that all LAB used during the fermentation process meet the requirements of the Qualified Presumption of Safety assessment [24,26]. The final product is added to the standard feed without altering dietary patterns.

### 2.2. Experimental Design

**Figure 1:**
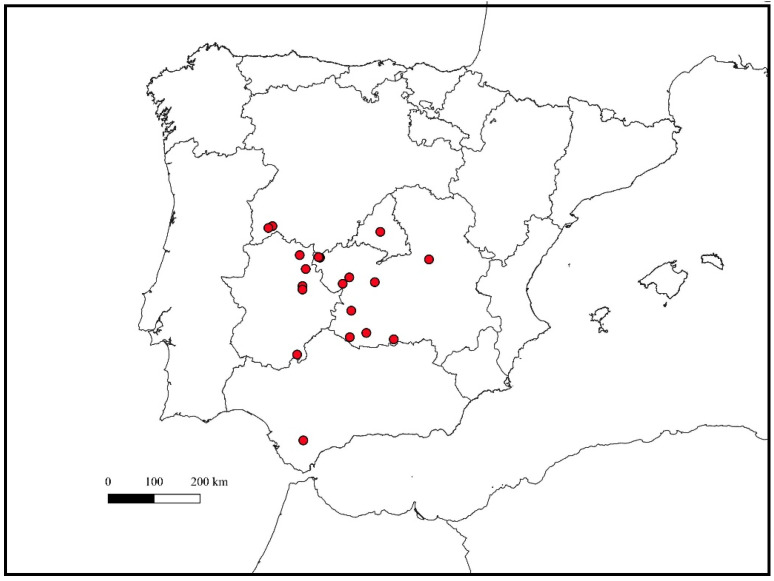
Geographical location of the 20 game estates in Spain included in this study.

This study was carried out in 20 game estates randomly divided into two groups—control estates (5) and supplemented estates (15)—all of which are located in mid-western Spain (Figure 1). These 20 estates are mainly focused on wild boar management and fenced to prevent the dispersion of wild boar as well as to avoid the entrance of new animals. Supplementary feed exclusively formulated for wild boar is provided in all these estates through selective feeders (only wild boar can enter) for at least 6 months per year.

Wild boar from 15 supplemented estates were fed with standard feed containing 3 g per kilogram of the postbiotic product during 30 consecutive days in a range of time compressed between 1st July and 1st September 2018. This ensured that most of the animals were frequenting the feeders, since this is the season of the year in which natural feed is scarce [14]. During this period, wild boar did not have access to any other supplementary food sources. The remaining five estates formed the control group, where wild boar were fed with standard food without postbiotic addition.

The epidemiological situation of wild boar from the control and supplemented estates was assessed during hunting events held from October 2017 to February 2018—pre-supplementation—and from October 2018 to February 2019—post-supplementation. This experimental design allowed us to evaluate the TB situation (the presence of TB gross lesions, the severity of TB lesions and seropositivity against *M. bovis*) in the control and supplemented estates, before and after postbiotic supplementation; hence, it is useful in evaluating the effect of this product on TB development.

### 2.3. TB Pathological Assessment

During this experiment (October 2017–February 2019), a total of 3449 wild boar were hunted during 51 hunting events conducted on selected game estates (Supplementary Table S1). To avoid biased data collection, at least 10 wild boar—when possible—from each hunting event were randomly selected, categorized by dental eruption pattern into two age classes—young (6 months to 12 months) and adult (more than 12 months) [27,28]—and inspected in order to assess the presence of TB-like lesions (TBLLs). A complete post-mortem examination was performed on 409 wild boar, targeting the main lymph nodes—submandibular, retropharyngeal, tracheobronchial, mediastinal, gastrohepatic and mesenteric—and thoracic and abdominal cavities and organs, namely, the lungs, liver, spleen. Positive wild boar were classified according to the severity of TBLLs—a “localized” pattern affecting only cephalic or mesenteric lymph nodes and a “generalized” pattern affecting at least two different organs or lymph nodes from different regions. TB diagnosis based on the presence of macroscopic lesions in carcasses is the officially recognized inspection used by Spanish Sanitary Authorities and it is considered an approved method for estimating TB prevalence [7].

### 2.4. TB Serological Survey

A serological survey was carried out in 18 (14 supplemented + 4 control) out of the 20 game estates included in the study in order to detect antibodies against *M. bovis*. During each hunting event, blood from selected wild boar was collected by puncturing the retro-orbital cavernous sinus, refrigerated at 4 °C and transported to the laboratory, where sera were extracted by centrifugation at 3000 rpm for five minutes and kept at −21 °C until processing.

To assess the presence of antibodies against *M. bovis* in wild boar sera, a commercial ELISA kit validated for wild boar (INGEZIM TB PORCINA, INGENASA, Madrid, Spain) was used following the manufacturer’s recommendations.

### 2.5. Statistical Analysis

The proportion of wild boar presenting with TBLLs, the percentage of animals with severe TBLLs and seroprevalence were the parameters used to study the TB situation in wild boar. All parameters were compared between control and supplemented populations at two time points—pre-supplementation and post-supplementation—to assess differences before and after the postbiotic supplementation. Intragroup differences were also assessed at two time points in order to evaluate the effect of postbiotics on supplemented populations and to detect possible variations in TB situation in control populations. All comparisons were tested using a Chi-square test, with a 5% level of significance.

Intragroup differences were further analyzed by dividing the supplemented population into two subsets: young animals and adults. This allowed us to distinguish a group of animals with recent infections—young animals aged between 6 and 12 months—and avoid misunderstanding the presence of chronic TBLL and seropositivity in animals who had developed the disease over a long period, as these symptoms are more likely to be present in adult wild boar aged more than 12 months.

## 3. Results

### 3.1. TB Pathological Evaluation

During pathological inspections, TBLLs were observed in 146 out of the 409 inspected wild boar (35.69%). Among the TB-affected animals, 32 wild boar showed TBLLs in multiple organs, resulting in a 27.82% rate of generalized TB patterns (32 out of 115). A complete pathological assessment could not be conducted in 31 wild boar displaying TBLLs, due to the lack of key organs at the moment of inspection—owing to shooting or dog bites—or because they were not eviscerated—owing to veterinary confiscation for TBLLs in cephalic lymph nodes. Information regarding the number of animals analyzed and the results of pathological assessment for each hunting event can be found in Supplementary Table S1.

Results obtained in the pathological evaluation of control and supplemented populations are summarized in Table 1. The TBLL rate found in animals belonging to control populations was similar to those found among supplemented animals in the pre-supplementation season (47.45% vs. 39.35%, χ^2^ = 0.84, *p*-value = 0.35). However, for the post-supplementation season, the TBLL rate was significantly higher in control wild boar compared to supplemented wild boar (45.23% vs. 24.84%, χ^2^ = 5.68, *p*-value = 0.017). Conversely, the ratio of generalized patterns did not show statistically significant differences among groups throughout the study (25% vs. 29.51% in the pre-supplementation season, χ^2^ = 0.40, *p*-value = 0.52 and 16.67% vs. 41.18% in the post-supplementation season, χ^2^ = 1.99, *p*-value = 0.32).

Regarding intragroup comparisons, animals belonging to control groups did not show significant differences between pre-supplementation and post-supplementation seasons in TBLL rate, nor in the ratio of generalized patterns. On the contrary, a lower TBLL rate was observed among supplemented animals in the post-supplementation season compared to the pre-supplementation season (24.84% vs. 39.35%, χ^2^ = 6.7/96, *p*-value = 0.01), while no differences were detected in the percentage of generalized patterns (41.18% vs. 29.51%, χ^2^ = 0.38, *p*-value = 0.53) among the same group of animals.

### 3.2. TB Serology

Results obtained in the serological tests revealed that 153 out of the 449 analyzed sera (34.1%) contained antibodies against *M. bovis.* The percentages of seropositive wild boar in control and supplemented estates were similar in the pre-supplementation season (45.59% vs. 35.48% χ^2^ = 1.63, *p*-value = 0.2). However, after supplementation, seropositivity was significantly lower in the supplemented group (48.61% vs. 22.73%, χ^2^ = 17.26, *p*-value = 3.24 × 10^−5^). Furthermore, whereas seropositivity did not change throughout the experiment in the control group (45.59% vs. 48.61%, χ^2^ = 0.26, *p*-value = 0.6), we observed a reduction in the percentage of seropositive animals in supplemented estates (35.48% vs. 22.73%, χ^2^ = 5.48, *p*-value = 0.019).

### 3.3. Assessing the Effect of Postbiotics in Different Age Groups

TB parameters obtained in each age class—young and adult—of the supplemented population are summarized in Table 2. In adults, no significant differences were detected when comparing TB parameters from the pre-supplementation and post-supplementation seasons. However, in young animals, the rates of TBLLs (χ^2^ = 9.9, *p*-value = 0.002) and seropositivity (χ^2^ = 6.64, *p*-value = 0.009) were significantly lower after supplementation compared to the previous season.

## 4. Discussion

The use of beneficial microbes is emerging in the field of wildlife disease mitigation [29,30]. To the extent of our knowledge, this is the first report describing oral supplementation with metabolites produced by beneficial bacteria—postbiotics—aimed at controlling infectious diseases in wild boar. Our results are suggestive of a beneficial effect of the postbiotic product on TB disease development in this species.

Our clinical trial is considered warranted and well validated for its intended purpose, since some requirements have been met that allow us to conclude a positive effect. Accordingly, no differences between groups were found before the study. On the contrary, while the TB situation in the control group remained stable, TB parameters after supplementation were reduced in the supplemented group.

In this study, the prevalence of gross TBLLs and TB seropositivity decreased by 36.87% and 35.94%, respectively, after oral supplementation with the postbiotic product in supplemented populations. Additionally, these parameters were significantly lower in supplemented estates compared to control estates after supplementation (a 45.08% and 53.24% reduction, respectively). These findings suggest a positive effect of the postbiotic supplementation on the TB situation, comparable with results obtained using other, previously tested measures.

The main strategies for controlling transmissible diseases shared with wildlife include preventive measures, host population control and vaccination [31]. Some of the vaccination strategies carried out under similar conditions reported comparable outcomes. Heat-inactivated vaccines decreased TBLLs by 34% [5] and 43.3% [32] when administered orally and 43.3% [32] and 66% [33] when administered parenterally, whereas oral BCG vaccination confers variable protection with divergent results [5,32,33]. Other studies with similar approaches yielded mixed results. While random culling decreased TBLLs and TB seroprevalence by 21–48% [34], selective culling failed to reduce TB seroprevalence [31]; other similar studies suggest that removal strategies should be implemented with complementary measures to observe any effect on TB prevalence [7,35].

In this study, the prevalence of generalized lesions remained stable in both groups before and after supplementation, in contrast to other similar studies [6,14]. This suggests that supplementation with postbiotics is more effective in preventing new infections than in reducing the severity of TB lesions.

When we explored the effect of postbiotics on young animals to distinguish between early and chronic infections, we observed that the prevalence of TBLLs and seropositivity dropped by 64.72% and 81.80% after supplementation, confirming a decrease in new contagions in young animals after consumption of postbiotics. Interestingly, these differences were not detected in adults. This could be due to the chronic evolution of the disease, since positive adult wild boar were most certainly infected before the experiment. Thus, we would need a long-term supplementation period to detect any effect on adult animals.

Once the host is infected by *M. bovis*, postbiotic metabolites may contain the spread of the disease through their effect on the viability of the bacteria and their interaction with the host immune system [16]. The registered postbiotic product used in this study (INGUBAL^®^, Ingulados S.L., Cáceres, Spain) consists of a postbiotic supplement that comprises fermentation metabolites including bacteriocins, chemical compounds and cell wall elements from lactic acid bacteria, as previously published [16]. Beneficial bacteria from which the supplement is produced are capable of locally secreting two-peptide bacteriocins—among other metabolites, including organic acids like lactate and acetate—when the bacteria reach the intestine, exerting an antimicrobial synergistic effect [16]. Due to these antimycobacterial properties, orally administered postbiotics may decrease the mycobacterial load in the gastrointestinal tract [16,36], thus reducing transmission from indirect fecal–oral contagion, given that fecal excretion is one of the main routes of *M. bovis* shedding in wild boar [37,38]. On the other hand, some of the cell wall components released after bacterial lysis may act as postbiotic immunomodulators, such as the collagen adhesin protein previously described [16]. In previous studies, we reported that LAB may influence mycobacteria phagocytic response, which is crucial for the survival of bacteria in the host [39]. Postbiotics could therefore act as innate immunomodulators antagonizing mycobacteria phagocytosis, provided that adhesin proteins compete for binding C-type lectin receptors and the classical C1-dependent complement activation pathway. Other cell wall and cytoplasmatic membrane elements that stimulate macrophage activation via Toll-like Receptors (TLRs) may also be present [16]. In this particular case, lipoteichoic acids (LTAs) and wall teichoic acid (WTA) are capable of activating macrophages via TLR2 and fimbrial proteins via TLR5 [40,41]. These postbiotic elements may trigger the host immune system, influencing the outcome of bovine TB. It has also been reported that postbiotic metabolites from lactobacilli increase levels of T helper type 1 (Th1) cytokines and decrease Th2-associated cytokines [21,23]. This immunomodulatory effect could also be positive for the greater control of bovine TB development, since Th1 response is essential for this purpose [39,42].

The postbiotic product is considered safe for use as feed supplement in wild boar provided that all LAB used during the fermentation process meet the requirements of the QPS [16]. It is also in accordance with the Regulation (EU) 2016/429 of The European Parliament and of the Council of 9 March 2016 on transmissible animal diseases. This measure is particularly feasible in managed populations living in fenced estates to control the dissemination of bovine TB at a specific hotspot of disease, but it could also be useful in estates that implement occasional supplementation, provided that this is time-controlled supplementation. The use of postbiotic supplements in wildlife represents as an alternative to other measures that could be controversial, such as culling—since wild boar have economic and cultural value in Spain—and measures that require considerable management of the animals—such as parenteral vaccination. Notwithstanding, this measure might foreseeably be considered a complementary tool in global eradication schemes of the disease on a larger scale.

The use of postbiotics to control bovine TB could also be extended to other maintenance hosts that limit the effectiveness of eradication programs. Thus, it could be tested in populations of red deer (*Cervus elaphus*) and fallow deer (*Dama dama*) in Spain, Eurasian badger (*Meles meles*) in the United Kingdom, white-tailed deer (*Odocoileus virginianus*) in North America, brush-tailed possums (*Trichosurus Vulpecula*) in New Zealand or African buffalo (*Syncerus caffer*) in South Africa [7,20,43]. Furthermore, postbiotic supplementation could act as a sustainable and useful measure for the control of other important diseases in wildlife and domestic animals. A recent study has shown the effect of postbiotics on the immune response of rabbits against myxomatosis, suggesting a potential use as a preventive in wild rabbits [44].

We are currently optimizing our existing studies to better understand the time–dose effect relationship and implementing our method in other TB wild reservoirs. Additionally, we are also developing new projects aimed at monitoring immunomodulatory markers in order to gain more in-depth knowledge about the effect on the hosts’ immune system.

## 5. Conclusions

Postbiotic supplementation in wild boar reduced the prevalence of TB lesions and seropositivity in treated estates. These findings support the potential of postbiotics as a safe, feasible and sustainable tool for controlling TB in this species, offering a promising addition to broader TB eradication efforts.

## Figures and Tables

**Table 1:** Results of pathological evaluation and serological tests of control and supplemented groups from hunting seasons before and after supplementation.

Group			Pre-Supplementation ^a^	Post-Supplementation ^b^
Control group	TB macroscopic lesions	Positive animals (%)	28 (47.45%)	19 (45.23%)
		Total evaluated	59	42
	Generalized patterns	Positive animals (%)	5 (25%)	2 (16.67%)
		Total evaluated	25	12
	TB seropositivity	Positive animals (%)	31 (45.59%)	35 (48.61%)
		Total evaluated	68	72
Supplemented group	TB macroscopic lesions	Positive animals (%)	61 (39.35%)	38 (24.84%) *
		Total evaluated	155	153
	Generalized patterns	Positive animals (%)	18 (29.51%)	7 (41.18%)
		Total evaluated	61	17
	TB seropositivity	Positive animals (%)	55 (35.48%)	35 (22.73%) **
		Total evaluated	155	154

^a^ Pre-supplementation season: from October 2017 to February 2018. ^b^ Post-supplementation season: from October 2018 to February 2019. * Intergroup (*p*-value = 0.017) and intragroup (*p*-value = 0.01) significant differences. ** Intergroup (*p*-value = 3.24 × 10^−5^) and intragroup (*p*-value = 0.019) significant differences.

**Table 2:** Results by age group of pathological evaluation and serological tests of supplemented groups from hunting seasons before and after supplementation.

Group			Pre-Supplementation ^a^	Post-Supplementation ^b^
Young wild boar (6–12 months)	TB macroscopic lesions	Positive animals (%)	17 (47.22%)	12 (16.66%) *
		Total evaluated	36	72
	Generalized patterns	Positive animals (%)	9 (52.94%)	3 (37.50%)
		Total evaluated	17	8
	TB seropositivity	Positive animals (%)	7 (26.92%)	3 (4.90%) **
		Total evaluated	17 (47.22%)	12 (16.66%) *
Adult wild boar (>12 months)	TB macroscopic lesions	Positive animals (%)	44 (36.97%)	26 (32.09%)
		Total evaluated	119	81
	Generalized patterns	Positive animals (%)	9 (20.45%)	4 (44.44%)
		Total evaluated	44	9
	TB seropositivity	Positive animals (%)	45 (37.81%)	25 (33.78%)
		Total evaluated	119	74

^a^ Pre-supplementation season: from October 2017 to February 2018. ^b^ Post-supplementation season: from October 2018 to February 2019. * Significant differences (*p*-value = 0.002). ** Significant differences (*p*-value = 0.009).

## Data Availability

The data presented in this study can be obtained upon request from the corresponding author.

## Supplementary Materials

The following supporting information can be downloaded at https://www.mdpi.com/article/10.3390/pathogens13121078/s1, Table S1: Number of hunted and inspected animals and tuberculosis prevalence found in hunting events included in this experience.
